# Dengue and diabetes comorbidity: an emerging public health threat

**DOI:** 10.1093/inthealth/ihae089

**Published:** 2024-12-16

**Authors:** Donal Bisanzio, Cássia Fernanda Estofolete, Richard Reithinger

**Affiliations:** International Development Group, RTI International, Washington D.C., USA; Laboratório de Pesquisas em Virologia, FAMERP, CREATE-NEO, São José do Rio Preto, São Paulo, Brazil; International Development Group, RTI International, Washington D.C., USA

## Abstract

Dengue is of growing global public health concern. Diabetes is a significant risk factor for severe dengue and dengue-related mortality. Countries with the highest number of reported dengue cases are projected to experience a substantial increase in diabetes by 2050. This likely will result in an increased incidence of dengue–diabetes comorbidity, and, hence, in severe dengue and dengue-related mortality. Countries that are or will be affected by a high burden for both diabetes and dengue should urgently design strategies to minimize the health and economic impact that a diabetes–dengue comorbidity could have on affected populations.

## Introduction

In the past 8 mo, the number of dengue cases reported globally has reached concerning levels. Thus, in Latin America, 11 517 728 suspected and 6 166 278 laboratory-confirmed cases were reported from 1 January to 18 August 2024; Brazil is experiencing the worst dengue outbreak ever recorded, with 382 315 cases reported from 8 April to 14 April alone.^1^ Multiple studies are forecasting heightened dengue transmission in currently endemic countries, as well as dengue spreading into currently non-endemic countries during the next two decades, driven by the expansion of the *Aedes* vector distribution range, increased urbanization and variations in temperature and precipitation due to climate change.^2^

The clinical symptomatology of dengue—ranging from mild, flu-like symptoms to haemorrhagic fever, dengue shock syndrome and death—can be substantially affected by chronic and immunocompromising diseases such as diabetes.^2^ Indeed, recent meta-analyses showed diabetes being a significant risk factor for severe dengue and dengue-related mortality.^3–5^ Thus, in their systematic review and meta-analysis on clinical predictors of severe dengue, Tsheten et al.^3^ showed that patients with pre-existing diabetes were almost at a threefold (OR 2.88; 95% CI 1.72 to 4.81) higher risk of developing severe dengue than patients without diabetes. In their systematic review and meta-analysis of risk factors for mortality in patients with dengue, Chagas et al.^4^ showed that patients with diabetes had an almost four times (OR 3.698, 95% CI 1.196 to 11.433) greater risk of dying than patients without diabetes. Both these findings were confirmed in a recent review by Lu et al.,^5^ who showed that a diagnosis of diabetes was associated with an increased risk for severe clinical presentations of dengue fever (OR 3.39; 95% CI 2.46 to 4.68), as well as a significantly increased risk of dengue-related death (OR 1.95; 95% CI 1.09 to 3.52).

## Global diabetes projections

Diabetes has become a major challenge to public health and healthcare systems worldwide, and it has been identified by the WHO as one of three target diseases in its WHO Global Action Plan for the Prevention and Control of Non-communicable Diseases.^6^ As per the 2023 World Health Statistics Annual Report,^7^ diabetes causes an estimated 2 million (95% uncertainty interval [UI] 1.4–2.7 million) deaths per year. Indeed, it was recently estimated that 529 million (95% UI 500–564 million) people are affected by diabetes, and that this number will surpass 1 billion people (1310 million; 95% UI 1220–1390 million) by 2050.^8^ The main reasons for this expansion are urbanization linked to broader macroeconomic and sociodemographic trends, and concurrent major behavioral shifts and changes in food systems and intake.^8^

**Figure 1.**
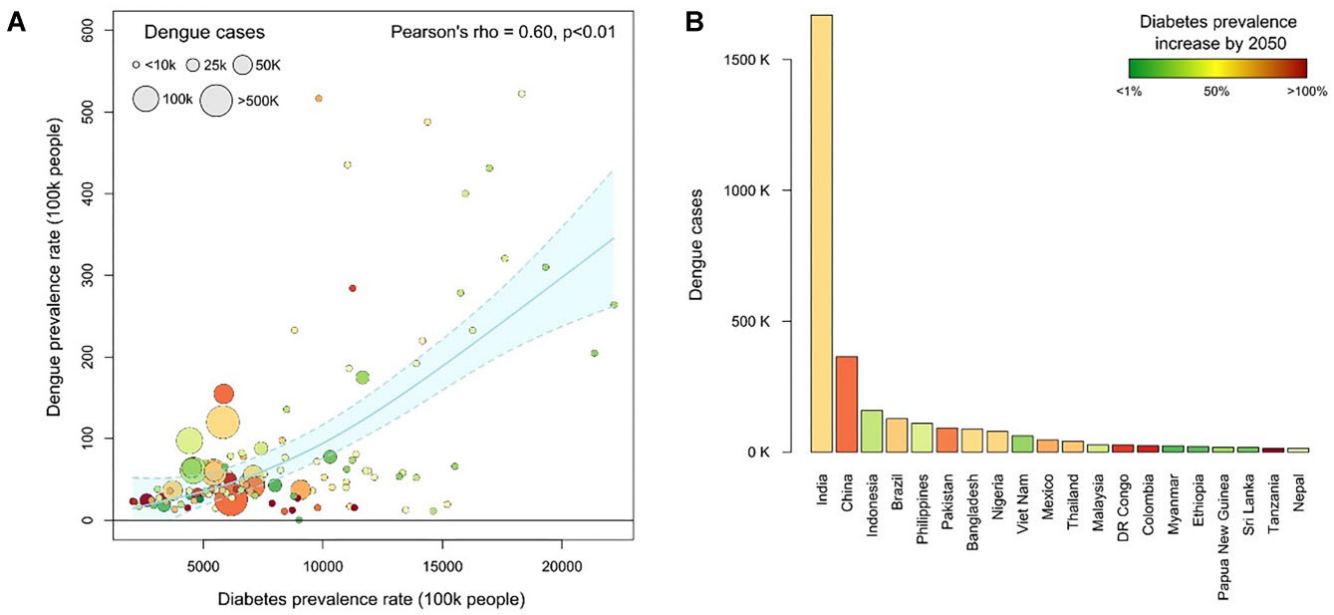
Comparison of symptomatic dengue prevalence (as per GBD 2019) and diabetes prevalence (as per GBD 2021) per 100 000 people. (A) compares the estimated prevalence rates of dengue and diabetes in all endemic countries^9^; bubbles represent absolute numbers of predicted dengue cases in 2019, as reported by the GBD 2021.^8^ Visualizations and statistical analyses were conducted in R-programming language (R Foundation for Statistical Computing, Vienna, Austria; https://www.R-project.org/); specifically, a Pearson's correlation coefficient test was carried out to test for the correlation between the two datasets. (B) illustrates the projected percentage changes in diabetes prevalence by 2050 for the 20 countries with the highest number of predicted dengue cases in 2019, as reported by GBD 2021.^8^ GBD: Global Burden of Disease.

To identify geographic areas where diabetes and dengue comorbidity may be highest, and, hence, diabetes-induced severe dengue and dengue-related mortality would be greatest, we compared the 2019 Global Burden of Disease study reported symptomatic dengue prevalence (per 100 000 people)^9^ with the recently reported and projected diabetes prevalence.^8^ We show a high degree of correlation: the 20 countries with the highest number of reported symptomatic dengue cases are projected to experience a median increase in diabetes cases of 61.8% (IQR 35.4–78%) by 2050 (Figure 1).

## Burden of comorbidity and policy implications

How diabetes mellitus affects the pathophysiology of dengue is an area of ongoing research, but it appears to involve glycemic control and platelet activity. Thus, hyperglycemia in patients with diabetes is thought to lead to immune response dysfunction, suppression of cytokine production, defective phagocytosis and immune cell dysfunction, in addition to the risk of natural barrier impairment due to neuropathy.^10^ Platelet activity is increased in patients with diabetes, inhibiting the interferon pathway and enhancing dengue virus replication.^11^

Given the projected increase of diabetes in dengue-endemic countries, health practitioners and policymakers should be aware that such an increase in diabetes is likely going to result in an increase of dengue–diabetes comorbidity, and, consequently, of severe dengue and dengue-related mortality. Similarly, health practitioners and policymakers in countries with an already high prevalence of diabetes and emerging dengue transmission should be cognizant that diabetes may exacerbate dengue symptoms. Actions to be taken in both sets of countries could include updating relevant clinical diagnosis and treatment guidelines to manage comorbid patients; sensitizing patients with diabetes to the risk and clinical signs of dengue, peak dengue transmission seasons and the heightened risk of potential adverse dengue outcomes; or facilitating access to vector control measures (e.g. spatial repellents, or indoor residual spraying) in households with a patient with diabetes. While some of the above actions have been taken, actions have been uneven in dengue-endemic and non-endemic countries. Thus, for example, the 2024 U. S. Centers for Disease Control Dengue Clinical Management Pocket Guide^12^ and Bangladesh's 2022 Pocket Guideline for Dengue Case Management^13^ mention diabetes as one of the coexisting conditions that are criteria for possible hospitalization, but do not provide further details on how to manage and monitor those patients. By contrast, India's 2023 National Guidelines for Clinical Management of Dengue Fever^14^ and the Pan American Health Organization's Guidelines for Patient Care in the Region of the Americas^15^ not only specify diabetes as a comorbidity, but also include specific sections describing how patients with diabetes should be diagnosed, treated and monitored (e.g. with insulin therapy).

Countries that currently have or are projected to have a high burden for both diabetes and dengue should recognize the possible threat that dengue–diabetes comorbidity represents, and design strategies to reduce the burden of both diseases to minimize the health and economic impact that a dengue–diabetes comorbidity could have on affected populations.

## Data Availability

Data to create Figure 1 are available from the authors upon request.
